# International severe asthma registry (ISAR): protocol for a global registry

**DOI:** 10.1186/s12874-020-01065-0

**Published:** 2020-08-14

**Authors:** J. Mark FitzGerald, Trung N. Tran, Marianna Alacqua, Alan Altraja, Vibeke Backer, Leif Bjermer, Unnur Bjornsdottir, Arnaud Bourdin, Guy Brusselle, Lakmini Bulathsinhala, John Busby, Giorgio W. Canonica, Victoria Carter, Isha Chaudhry, You Sook Cho, George Christoff, Borja G. Cosio, Richard W. Costello, Neva Eleangovan, Peter G. Gibson, Liam G. Heaney, Enrico Heffler, Mark Hew, Naeimeh Hosseini, Takashi Iwanaga, David J. Jackson, Rupert Jones, Mariko S. Koh, Thao Le, Lauri Lehtimäki, Dora Ludviksdottir, Anke H. Maitland-van der Zee, Andrew Menzies-Gow, Ruth B. Murray, Nikolaos G. Papadopoulos, Luis Perez-de-Llano, Matthew Peters, Paul E. Pfeffer, Todor A. Popov, Celeste M. Porsbjerg, Chris A. Price, Chin K. Rhee, Mohsen Sadatsafavi, Yuji Tohda, Eileen Wang, Michael E. Wechsler, James Zangrilli, David B. Price

**Affiliations:** 1 The Institute for Heart Lung Health, Vancouver, Canada; 2 AstraZeneca, Gaithersburg, USA; 3 Department of Pulmonary Medicine, University of Tartu and Lung Clinic, Tartu University Hospital, Tartu, Estonia; 4 Center of Physical Activity Research, Rigshospitalet and Copenhagen University, Copenhagen, Denmark; 5 Department of Respiratory Medicine & Allergology, Skåne University Hospital, Lund, Sweden; 6 Faculty of Medicine, University of Iceland, Reykjavik, Iceland; 7 Department of Respiratory Diseases, Montpellier University Hospitals, Hopital Arnaud de Villeneuve and PhyMed Exp (INSERM U 1046, CNRS UMR9214), Universite de Montpellier, Montpellier, France; 8 Department of Respiratory Medicine, Ghent University Hospital, Ghent, Belgium; 9 Departments of Epidemiology and Respiratory Medicine, Erasmus Medical Center Rotterdam, Rotterdam, The Netherlands; 10 Optimum Patient Care, Cambridge, UK; 11 Centre for Public Health, Queen’s University Belfast, Belfast, UK; 12 Personalized Medicine Asthma & Allergy Clinic, Humanitas University & Research Hospital, Milan, Italy; 13 SANI-Severe Asthma Network Italy, Milan, Italy; 14 Division of Allergy, Department of Medicine, Asan Medical Center, College of Medicine, University of Ulsan, Seoul, South Korea; 15 Faculty of Public Health, Medical University – Sofia, Sofia, Bulgaria; 16 Son Espases University Hospital-IdISBa-Ciberes, Mallorca, Spain; 17 Clinical Research Centre, Smurfit Building Beaumont Hospital and Department of Respiratory Medicine, RCSI, Dublin, Ireland; 18 Australasian Severe Asthma Network, Priority Research Centre for Healthy Lungs, University of Newcastle, Newcastle, Australia; 19 Department of Respiratory and Sleep Medicine, Hunter Medical Research Institute, John Hunter Hospital, New Lambton Heights, Australia; 20 Centre for Experimental Medicine, Queen’s University Belfast, Belfast, UK; 21 Alfred Health & Monash University, Melbourne, Australia; 22 Department of Respiratory Medicine & Allergology, Faculty of Medicine, Kindai University Hospital, Ōsakasayama, Japan; 23 Guy’s & St Thomas’ NHS Trust and King’s College London, London, UK; 24 Faculty of Medicine & Dentistry, University of Plymouth, Plymouth, UK; 25 Department of Respiratory & Critical Care Medicine, Singapore General Hospital and Duke-National University Singapore Medical School, Singapore, Singapore; 26 Allergy Centre, Tampere University Hospital and Tampere University, Tampere, Finland; 27 Department of Respiratory Medicine, Faculty of Medicine, Landspitali University Hospital and University of Iceland, Reykjavik, Iceland; 28 University of Amsterdam, Amsterdam, The Netherlands; 29 Royal Brompton & Harefield NHS Foundation Trust, London, UK; 30 University of Athens, Athens, Greece; 31 University of Manchester, Manchester, UK; 32 Pneumology Service, Hospital Universitario Lucus Augusti, Lugo, Spain; 33 University of Sydney Medical School, Sydney, Australia; 34 UK Severe Asthma Network, Barts Health NHS Trust and Queen Mary University of London, London, UK; 35 University Hospital “Sv. Ivan Rilski”, Sofia, Bulgaria; 36 Bispebjerg Hospital, Copenhagen University, Copenhagen, Denmark; 37 The Catholic University of Korea, Seoul, South Korea; 38 Faculty of Pharmaceutical Sciences, University of British Columbia, Vancouver, Canada; 39 Division of Allergy & Clinical Immunology, Department of Medicine, National Jewish Health and Division of Allergy & Clinical Immunology, Department of Internal Medicine, University of Colorado Hospital, Denver and Aurora, CO USA; 40 Division of Pulmonary, Critical Care and Sleep Medicine, Asthma Program, National Jewish Health, Denver, USA; 41 Observational and Pragmatic Research Institute, Singapore, Singapore; 42 Academic Primary Care, Division of Applied Health Sciences, University of Aberdeen, Polwarth Building, Foresterhill, Aberdeen, AB25 2ZD UK

**Keywords:** Disease registry, Protocol, Real-world, Severe asthma

## Abstract

**Background:**

Severe asthma exerts a disproportionately heavy burden on patients and health care. Due to the heterogeneity of the severe asthma population, many patients need to be evaluated to understand the clinical features and outcomes of severe asthma in order to facilitate personalised and targeted care. The International Severe Asthma Registry (ISAR) is a multi-country registry project initiated to aid in this endeavour.

**Methods:**

ISAR is a multi-disciplinary initiative benefitting from the combined experience of the ISAR Steering Committee (ISC; comprising 47 clinicians and researchers across 29 countries, who have a special interest and/or experience in severe asthma management or establishment and maintenance of severe asthma registries) in collaboration with scientists and experts in database management and communication. Patients (≥18 years old) receiving treatment according to the 2018 definitions of the Global Initiative for Asthma (GINA) Step 5 or uncontrolled on GINA Step 4 treatment will be included. Data will be collected on a core set of 95 variables identified using the Delphi method. Participating registries will agree to provide access to and share standardised anonymous patient-level data with ISAR. ISAR is a registered data source on the European Network of Centres for Pharmacoepidemiology and Pharmacovigilance. ISAR’s collaborators include Optimum Patient Care, the Respiratory Effectiveness Group (REG) and AstraZeneca. ISAR is overseen by the ISC, REG, the Anonymised Data Ethics & Protocol Transparency Committee and the ISAR operational committee, ensuring the conduct of ethical, clinically relevant research that brings value to all key stakeholders.

**Conclusions:**

ISAR aims to offer a rich source of real-life data for scientific research to understand and improve disease burden, treatment patterns and patient outcomes in severe asthma. Furthermore, the registry will provide an international platform for research collaboration in respiratory medicine, with the overarching aim of improving primary and secondary care of adults with severe asthma globally.

## Background

Treatment of severe asthma is challenging because of the heterogeneous nature of the disease, which comprises various phenotypes and endotypes [1–3]. The heterogeneity of airway inflammation in patients with severe asthma makes profiling of patients considerably important toward identifying “treatable traits” [4]. Moreover, while severe asthma affects approximately 5% − 15% of all patients with asthma [5–7], the associated impact on the lives of patients and caregivers is disproportionately high [8, 9]. Inadequately controlled severe asthma is also associated with poor clinical outcomes and a high economic burden [9–12].

National and local asthma registries are a source of real-world data for asthma management and may contain data at the patient, provider and clinic levels [13–17]. However, because of considerable differences in the definitions of severe asthma [5, 18–20] and country- and region-specific differences in access to health care [21, 22], patient populations included in these registries may be heterogeneous in terms of demographics, clinical characteristics, inflammatory phenotypes, captured data and reported outcomes. Additionally, depending on the size of existing asthma registries, they may be limited by inadequate statistical power. As a result of this heterogeneity, interpretation of data across patient populations and geographies can be challenging. Therefore, a standardised global dataset is urgently needed to:

- retain all the values of local registries and connect them to enable and promote inter-operability, data sharing and cross-comparison;
- have sufficient statistical power to answer pertinent clinical and research questions;
- reduce the variability of data collected by standardising variables across countries and regions;
- have pre-defined and extensive processes in place to ensure that data capture and data harmonisation are of high quality;
- improve understanding of the severe asthma population and examine the response to therapies and other interventions as a function of nationality, phenotypes, biomarkers, current treatment and socio-economic status; and
- permit continued development with long-term patient follow-up to enable a real-life understanding of severe asthma.

**Fig. 1:**
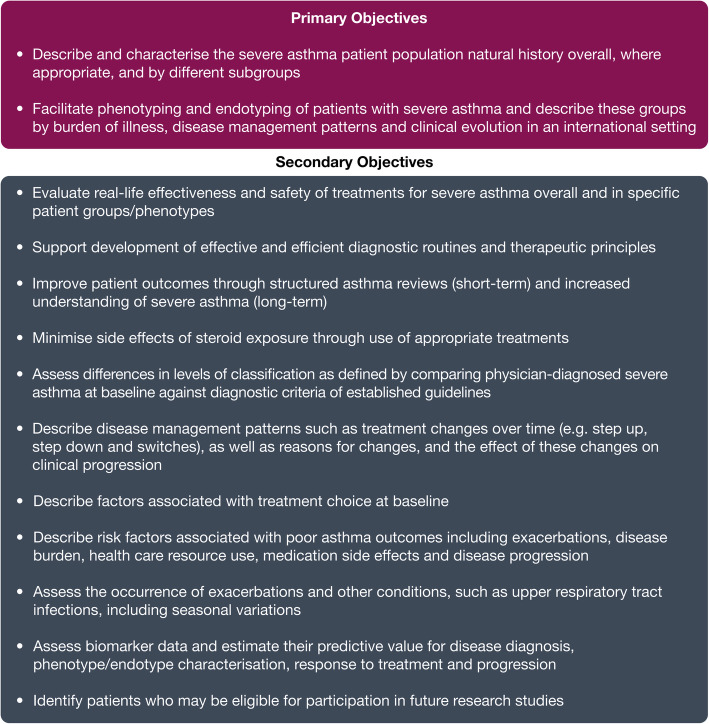
Objectives of the International Severe Asthma Registry

The International Severe Asthma Registry (ISAR) is a collaborative initiative comprising existing and new registries that builds on data from multiple nations and regions and increases the statistical power and comparability of data. It is the first global adult severe asthma registry to be established. The primary objectives of ISAR (Fig. 1) are to describe and characterise the natural history of the severe asthma patient population overall and by different subgroups and to facilitate phenotyping and endotyping of patients with severe asthma such that these patient groups can be described by burden of illness, disease management patterns and clinical evolution in an international setting. These objectives were developed to improve understanding of the clinical features and outcomes of severe asthma, with the overarching aim of improving the care of adults with severe asthma globally.

Here, we describe the ISAR protocol for registry development and management, the rationale behind each step of the process and the potential benefits of ISAR to adult patients with severe asthma.

## Methods

### Registry design and governance

ISAR, a multi-country, observational initiative, will both prospectively and retrospectively collect data on adult patients with severe asthma. A registered data source on the European Network of Centres for Pharmacoepidemiology and Pharmacovigilance (ENCePP) [23], ISAR is currently supported by three core collaborators: Optimum Patient Care (OPC), the Respiratory Effectiveness Group (REG) and AstraZeneca. OPC is a not-for-profit social enterprise providing medical research and services to improve the diagnosis, treatment and care of chronic diseases [24] and is responsible for ISAR database management, data processing and analyses. REG is an investigator-led, not-for-profit research initiative promoting the value of real-life research [25]. AstraZeneca and OPC are co-founders and joint sponsors of ISAR.

#### Oversight

**Fig. 2:**
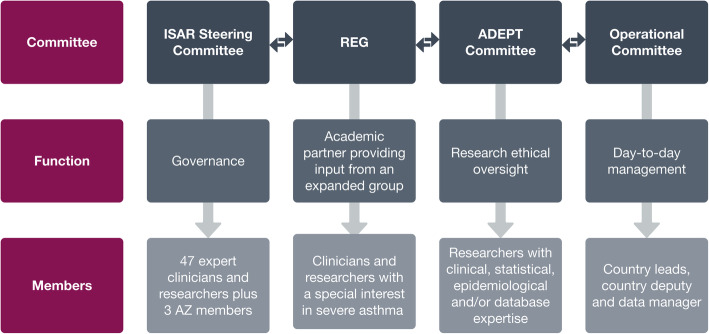
International Severe Asthma Registry governance. *ADEPT* Anonymised Data Ethics & Protocol Transparency, *AZ* AstraZeneca, *ISAR* International Severe Asthma Registry, *REG* Respiratory Effectiveness Group

ISAR is overseen by four governing bodies (Fig. 2): the ISAR Steering Committee (ISC), REG, the Anonymised Data Ethics & Protocol Transparency (ADEPT) Committee [26] and the ISAR Operational Committee.

The ISC comprises 47 clinicians and researchers across 29 countries in North America, South America, Europe, Asia, Middle East and Oceania, with a special interest in severe asthma and/or experience in establishing and maintaining a severe asthma registry. The ISC also includes members of OPC and medical experts from AstraZeneca. Since ISAR is an open registry, academic and commercial entities can register their interest to participate in ISAR and seek access to the data for research purposes, with all research proposals requiring approval by the ISC via a democratic voting process. Each member country and AstraZeneca have one vote on project selection, with OPC holding the casting vote in the event of ties. AstraZeneca does not vote on research proposals from commercial entities.

REG is the academic partner comprising over 420 experts in severe asthma and will provide academic oversight and support. The ADEPT Committee (commissioned independently by REG), using pre-specified criteria [24], will review and approve the scientific merit of all research proposals submitted to ensure scientific integrity, robustness and compliance with all relevant ethical considerations and to ensure that the database research is clinically appropriate and valuable to patients, public health and health care.

The ISAR Operational Committee includes participating country representatives (e.g. country lead, deputy and data managers) and will be involved in the day-to-day running of ISAR. The Operational Committee within each country is led by the country-specific ISC member; therefore, sound procurement of data is also partly ensured by the ISC.

### Registries, countries and experts

Existing registries were selected for possible collaboration via a systematic internet search of PubMed, MEDLINE, EMBASE, Google Scholar and Web of Science using broad search terms to identify all severe asthma registries. Registries for participation in ISAR were identified with the help of REG’s and OPC’s global network of leading respiratory scientists and severe asthma experts. These experts were either the lead of an existing registry, were linked with the lead or had previously expressed an interest in developing a severe asthma registry for their respective country. Leaders of existing and potential new severe asthma registries were approached and formally invited by ISAR to discuss collaboration and contribution to the ISAR initiative. Table 1 lists the existing and new registries collaborating with ISAR.

**Table 1:** List of existing and new registries collaborating with ISAR

Registry status	Collaborating country	Registry name	Start year
Existing Registry	UK	UK Severe Asthma Registry	2006
	USA	National Jewish Health Electronic Medical Record (NJH EMR)	2010
	South Korea	Severe Asthma Work Group of Korean Academy of Asthma, Allergy and Clinical Immunology (KAAACI)	2010
	Germany	German Asthma Network (GAN)	2011
	Australia & New Zealand	Australasian Severe Asthma Registry (ASAR) hosted by TSANZ	2013
	Ireland	INhaler Compliance Assessment in Severe Unstable Asthma (INCA SUN)	2015
	Italy	Severe Asthma Network Italy (SANI)	2016
	Spain	Spanish Guideline on the Management of Asthma Database (GEMA-Data)	2017
New Registry	Denmark	Danish Severe Asthma Registry (DSAR)	2018
	Sweden	Swedish Severe Asthma Registry	Starting in 2020
	Finland	Currently collecting data independently from ISAR	2019
	Iceland	Currently collecting data independently from ISAR	2020
	Norway		Starting in 2021
	Bulgaria	Bulgarian Severe Asthma Registry (BULSAR)	2018
	Portugal	Portugal Severe Asthma Registry (Registo de Asma Grave Portugal [RAG])	2018
	Russia	Russian Severe Asthma Registry (RSAR)	2018
	Argentina	Argentinian Severe Asthma Registry	2019
	Belgium	Currently collecting data independently from ISAR	2018
	Brazil	Brazilian Severe Asthma Registry	Starting in 2020
	Canada	Canadian Severe Asthma Registry	2019
	China		Starting in 2021
	Colombia	Colombian Severe Asthma Registry	2019
	France	French Severe Asthma Registry	2019
	Greece	Greek Severe Asthma Registry	2019
	India	Indian Severe Asthma Registry	2019
	Japan	Japanese Severe Asthma Registry	2019
	Kuwait	Kuwaitian Severe Asthma Registry	2018
	Mexico	Mexican Severe Asthma Registry	2019
	Poland	Polish Severe Asthma Registry	2020
	Saudi Arabia	Saudi Arabian Severe Asthma Registry	2019
	Singapore	Singapore Severe Asthma Registry (S-SAR)	2020
	Taiwan	Taiwanese Severe Asthma Registry	2019
	UAE	UAE Severe Asthma Registry	2019

*ISAR* International Severe Asthma Registry, *UAE* United Arab Emirates, *UK* United Kingdom, *USA* United States of America

To facilitate global collaboration efforts, various aspects of the ISAR structure and deliverables, such as principles of data collection and sharing, as well as research prioritisation, will be jointly discussed between OPC and the lead entity for each registry/country and will be tailored towards each contributing country during the ISC meetings as well as country-specific meetings. The lead entity, depending on the local circumstances, could either be a university, a lead study site or the respective national thoracic society. In all instances, the lead entity is responsible for overseeing data collection, including combining data from any satellite sites, before making the country-wide data available to ISAR. In countries with no existing registries, this approach, in effect, creates a country-level registry that allows for the creation of a locally hosted central registry for the country’s combined data, which can be used to enhance local-level research, and provides longitudinal data that will help clinicians and patients better understand underlying phenotypes of severe asthma and enhance precision medicine.

The ethics application process differs among collaborating registries. Within some registries, ethics approval is required at each individual satellite site, whereas for other collaborators, a central ethics committee can process applications on behalf of all satellite sites.

In addition, the ISAR study group comprises core panel members of ISAR who will provide collective expertise, scientific knowledge and experience in database management and research (Additional file 1: Appendix).

### Patients

Patients in ISAR will be included from the participating existing and newly created local/regional registries. To be included, patients (≥18 years of age) should be receiving treatment according to the 2018 definitions of the Global Initiative for Asthma (GINA) Step 5 or should be uncontrolled on GINA Step 4 treatment [19, 27]. Uncontrolled asthma is defined as per the American Thoracic Society/European Respiratory Society criteria [5]. Detailed inclusion and exclusion criteria are listed in Table 2. These eligibility criteria were chosen to reflect severe asthma patients in the real-world setting and to broaden the scope to include patients with uncontrolled moderate-to-severe asthma. Notably, patients with a history of smoking are not excluded. In addition, patients with asthma-chronic obstructive pulmonary disease overlap (ACO), as defined in the 2018 GINA report [19], will be included. Informed consent will be obtained from patients where required to allow anonymised data sharing for approved research projects.

**Table 2:** ISAR patient inclusion and exclusion criteria

Inclusion	Exclusion
Adult (≥18 years old) patients with severe asthma	Lack of informed consent for participation
Undergoing GINA Step 5 treatment^a^ [19] or Uncontrolled on GINA Step 4 treatment [19] Uncontrolled defined as at least one of the following (per ATS/ERS guidelines [5]):	
Poor symptom control: ACQ consistently > 1.5, ACT < 20 (or ‘not well controlled’) [19]	
Airflow limitation: Pre-bronchodilator FEV_1_ < 80% predicted, with reduced FEV_1_/FVC (defined as less than the lower limit of normal)	
Serious exacerbations: ≥1 hospitalisation, ICU stay or mechanical ventilation in the previous year	
Frequent severe exacerbations: ≥2 bursts of systemic corticosteroids with each course > 3 days in the previous year	

^a^Asthma controlled on high-dose ICS/LABA treatment was not part of the current inclusion for ISAR as this treatment approach is not yet adopted by clinicians

*ACQ* Asthma Control Questionnaire, *ACT* Asthma Control Test, *ATS* American Thoracic Society, *ERS* European Respiratory Society, *FEV*_*1*_ forced expiratory volume in 1 s, *FVC* forced vital capacity, *GINA* Global Initiative for Asthma, *ICS* inhaled corticosteroids; *ICU* intensive care unit, *ISAR* International Severe Asthma Registry, *LABA* long-acting β_2_-agonist

On average, 2000 new patients will be enrolled globally each year, for at least 5 years from the start of ISAR (May 2017). Patients receiving care at severe asthma secondary and tertiary care centres in each participating country will be included, in accordance with local regulatory/ethics requirements. One follow-up per year is required with collaborating centres, which is in line with the minimum number of visits to a severe asthma specialist centre expected per patient. A minimum of two-thirds of patients enrolled within each participating registry are predicted to be retained for annual follow-up, after adjusting for attrition.

### Database

The ISAR initiative is a partnership with national and regional registries, such that each participating registry retains ownership of their own data, but shares their anonymised and de-identified data with ISAR for approved research purposes.

A collaboration and data sharing agreement will be negotiated and signed between OPC and the lead entity of each registry. The agreement will govern the:

- method of storage and transmission of data;
- data security and compliance with OPC data security standards;
- list of variables required to be extracted from each country-specific database;
- oversight (by OPC) to ensure confidentiality of the data received; and
- remote retrieval and appraisal of data from each country by OPC.

**Fig. 3:**
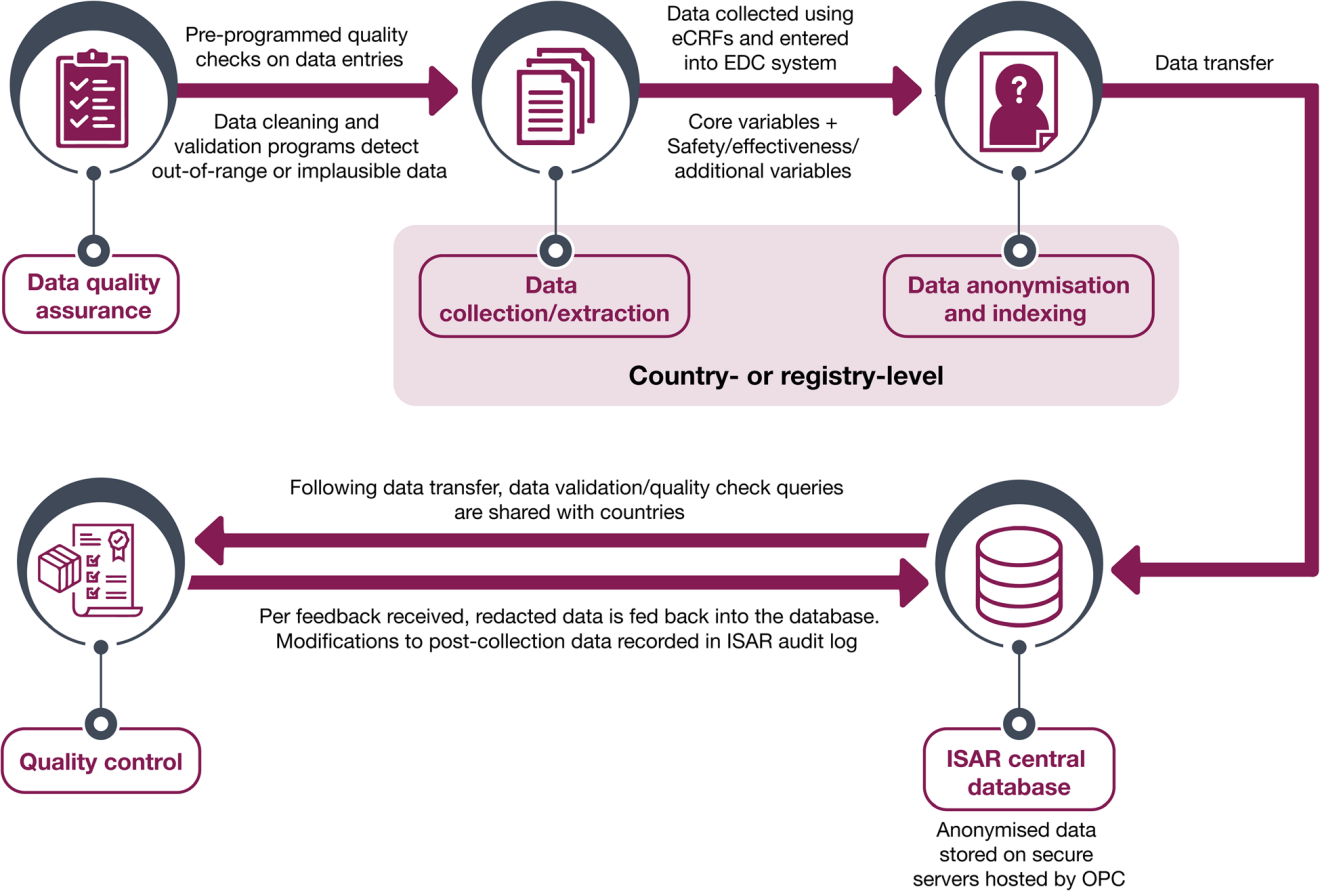
Steps involved in data collection, transfer and storage. *eCRF* electronic case report form, *EDC* electronic data capture, *ISAR* International Severe Asthma Registry, *OPC* Optimum Patient Care

The steps involved in data acquisition, quality control and management of ISAR have been outlined in Fig. 3.

#### Data collection

Data will be collected from a combination of existing and new registries, and each country will have a data collector trained in human subject research with relevant experience in Good Clinical Practice or country-specific equivalent guidelines. A small and limited financial incentive is also provided to compensate for any time spent on data entry by health care providers or allied workers. To accommodate the recently enacted European Union General Data Protection Regulation (EU GDPR) [28], and/or local ethics regulatory mandates, some countries will provide summarised statistics for research projects, while others will provide individual-level data in the initial stage of the ISAR collaboration. However, all registries (as required) plan and work with ethics committees to transfer individual-level data. ISAR will collect only anonymised data from all collaborating registries and ensure that the strictest security measures are in place for data sharing and hosting. A data transfer standard operating procedure (SOP) is provided to each registry to guide the anonymous and safe transfer of data.

**Fig. 4:**
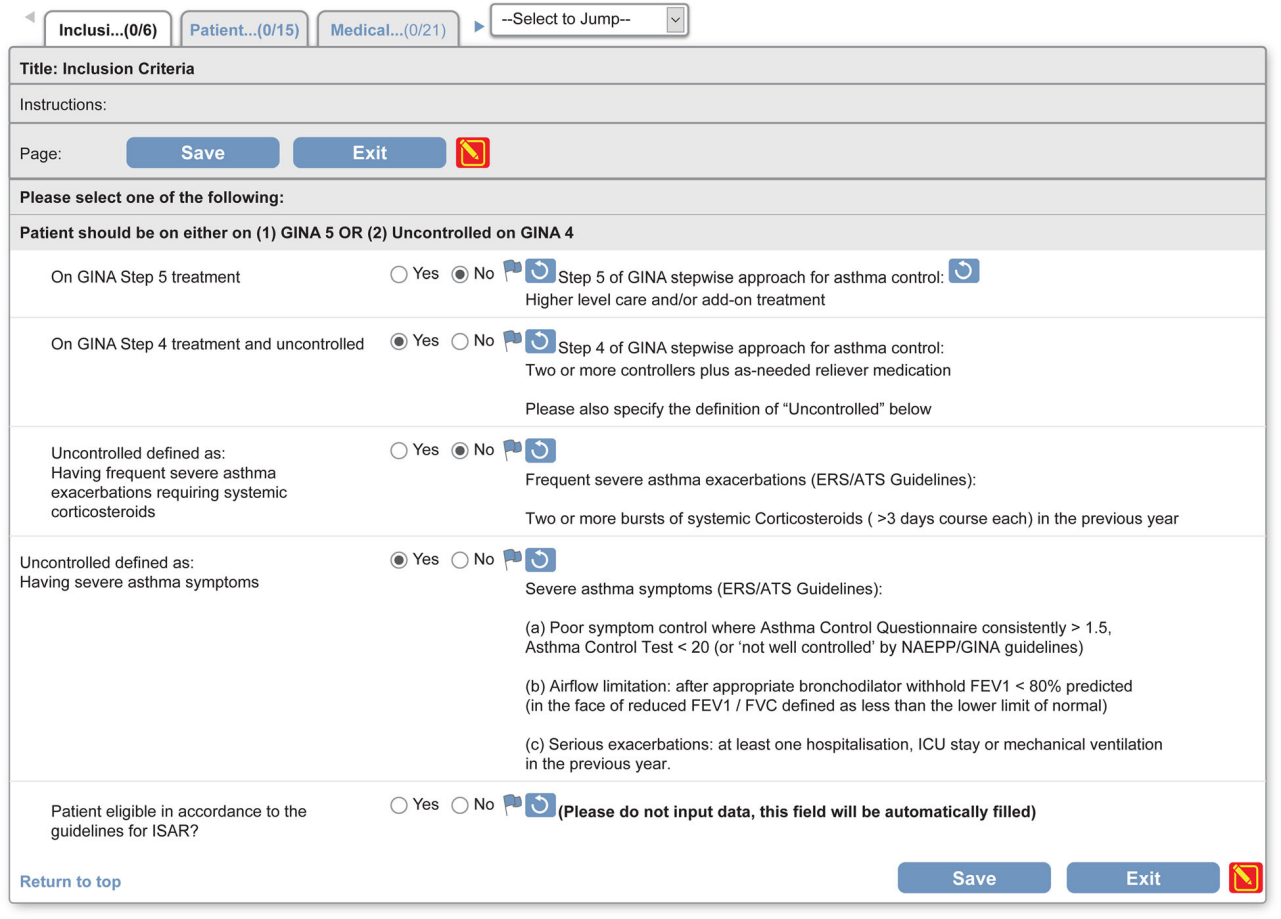
International Severe Asthma Registry electronic case report form (screenshot). *ATS* American Thoracic Society, *ERS* European Respiratory Society, *FEV1* forced expiratory volume in 1 second, *FVC* forced vital capacity, *NAEPP* National Asthma Education and Prevention Program

Data from the existing registries will be collected using existing systems (e.g. Dendrite Clinical Systems, United Kingdom [UK]; REDCap, Australia; Zitelab, Denmark) that are largely aligned with the standard data collection fields of ISAR. Data imported will be as per instructions listed in a separate ISAR data management plan, which will be provided to all registries. Data will be collected using a comprehensive electronic case report form (eCRF; screenshot in Fig. 4). Although registries can enter data directly in the eCRFs, they can also opt to collect the data on paper and enter it into the eCRF later, based on their clinical process. All eCRFs will be completed by designated personnel trained (via an on-site/remote training session) on data entry, and a data collection SOP will be provided with instructions on how to complete the CRF/eCRF, with a detailed explanation of the data fields. Data collection will comply with the standards established by the ISC and agreed by each participating registry, allowing datasets across all registries to be combined. These procedures are set in place to ensure uniform interpretation of variables across countries and cultures, further effectively standardising ISAR data.

#### Data quality and management

Functions, processes and specifications for data collection, extraction, delivery and cleaning are outlined in the ISAR data management plan. For instance, data quality is ensured before and during the data collection process through a series of pre-programmed data quality checks that automatically detect out-of-range or anomalous entries on the eCRF. Most of the fields requested on the ISAR eCRF are numeric or categorical to minimise data entry errors. After data extraction, further data cleaning and validation processes will also be performed on all data to maximise data quality control. Ad hoc queries, done at the country level or OPC level, will be generated within the electronic data capture (EDC) system and followed up with country data managers and/or the country study coordinator (where applicable) for resolution. All data modifications will be recorded in an audit log and all data transfers and disputes will be shared and documented in the country and ISAR central data manager logs.

#### Electronic data capture

All new data will be entered directly into the EDC system (REDCap or OpenClinica). Where feasible, patient data from electronic medical records will be integrated with the EDC systems to maximise data collection resources and reduce the time needed for data entry. Both new and existing data collection platforms will accommodate ISAR variables and anonymise and de-identify data prior to importing it to the central data warehouse, where the data will be stored with a unique patient identification number. The key code linking the unique identifier to the relevant patient will be held by the patient’s health care provider. ISAR will have no access to the linkage files. All participating sites will have access to their own data and will be trained on using the available online data capture systems. Physician and data entry personnel will be able to access their local EDC account with a username and password, with each user being prompted to change their unique password on a yearly basis.

OPC will be responsible for monitoring and mapping the data into the central ISAR data repository by using the Systematized Nomenclature of Medicine Clinical Terms (SNOMED CT) code [29] for standardised use globally. In addition, all existing registries using unique EDC systems will be responsible for extracting batches of patient data at a quarterly frequency for inclusion to ISAR. OPC will be responsible for safely transporting and importing each batch into the central ISAR data repository permitted by specific country’s data regulations.

For countries with data sharing regulations delimiting data privacy, such as the EU GDPR, ISAR will accommodate anonymised data sharing on a project-by-project basis. In these cases, the patient-level data are kept/hosted within the country and shared as needed for approved ISAR research.

#### Registry variables

A modified Delphi consensus–driven approach [30] was used to develop a standardised set of core and research variables for ISAR [31]. The ISC will hold regular meetings to ensure continued expert input throughout the development and expansion of the registry, in terms of the collaborators and investigators added, as well as data collected. Standardised data will be collected in three distinct fields:

##### Core variables

**Fig. 5:**
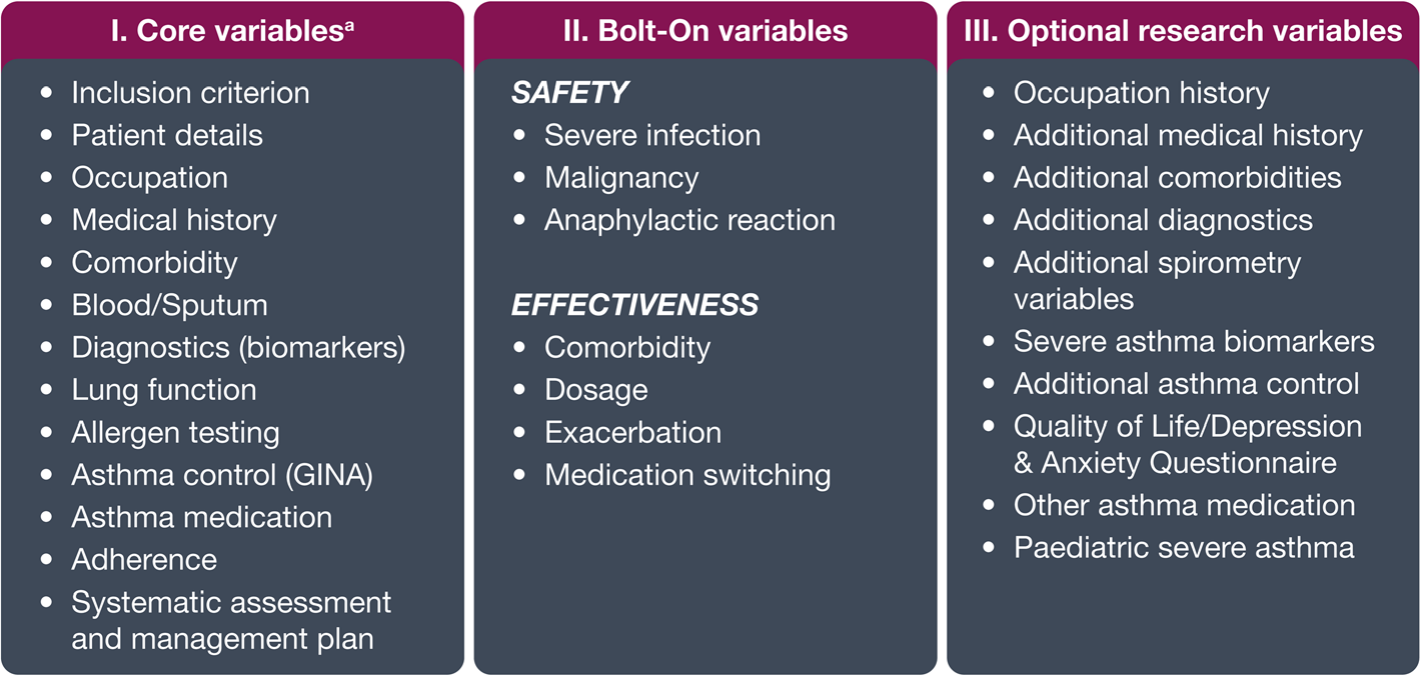
International Severe Asthma Registry variables. ^a^Collected by all participating registries. *GINA* Global Initiative for Asthma

These comprise 13 categories encompassing 95 core variables (Fig. 5; Additional file 2: Table S1), which include data on patient demographics, medical history and diagnostics, clinical characteristics, patient-reported outcomes and treatment management plans. These core variables are mandatory and should be collected by any registry wishing to contribute data to ISAR. Additional variables (e.g. those collected by the UK registry) can be reviewed by each ISAR member and included into the core variable list. However, no change is planned in the core variables for the first 5 years of ISAR. Each country is permitted to keep any number of these additional variables as extended variables in their own local registry/database.

##### Safety and effectiveness bolt-on variables

Safety variables (Fig. 5; Additional file 3: Table S2) comprising severe infections, malignancy and anaphylactic reactions were chosen because of evidence indicating the potential association between the use of biologics and these adverse events [32, 33]. Considering that these outcomes and associations have mostly been studied in small populations, the inclusion of these safety bolt-on variables will enable investigation and evaluation of these in a larger, global population with severe asthma.

Similarly, the effectiveness variables (Fig. 5; Additional file 3: Table S2) were selected to collect indications of osteoporosis, circulatory system diseases, ocular diseases (glaucoma and cataract), renal failure, type II diabetes mellitus, depression, anxiety, pneumonia, peptic ulcer, obstructive sleep apnoea and details on oral and inhaled corticosteroid doses, as well as reasons for medication therapy switches.

##### Additional research variables

These comprise all variables deemed useful for scientific research and maximal data collection, such as occupation, medical history, additional comorbidities, diagnostics, spirometry variables, biomarkers, asthma control, quality of life or depression and anxiety questionnaire, other asthma medications and paediatric severe asthma (Fig. 5; Additional file 4: Table S3). Extended variables will be collected via standardised bolt-on modules and will be available by choice to registries according to their preference. If resources allow, each registry is free to add additional variables of specific local interest, such as pregnancy, to their data collection tool.

#### Data ownership

Each country retains ownership of their data, and all participating countries agree to allow output of data from their respective registries upon joining ISAR for collaborative independent research approved by the ISC and ADEPT. The extraction and integration of datasets for ethically approved research studies will be managed by OPC, and the nature and frequency of data extraction and transfer (quarterly) from registries to OPC are detailed in the ISAR data sharing agreement.

#### Research

In terms of research output, the interim research goal of ISAR is to complete one global research project per year and to create four additional project-specific datasets for academic and commercial research by ISAR members. Non-core research activities will be presented as an additional two abstracts and two manuscripts per year from 2018 to 2021.

While ISAR will continue to actively seek out new partners, new collaborators may also join ISAR by using the ‘Join Us or Register Interest’ option on the ISAR home page (http://isaregistries.org/). Research ideas may be suggested by ISC members, country leads, and contributors and visitors to the ISAR website (which may include third party commercial and academic research organisations), by clicking the ‘Submit Proposal or Request Research’ tab on the ISAR home page. All research ideas will be reviewed, assessed and prioritised by the ISC. Notably, regardless of commercial partners or collaborators, there will be no inferential drug-to-drug comparison in ISAR; however, in the context of baseline patient characteristics and treatment patterns (e.g. switching), the proportions of patients on specific biologics may be described and comparisons of outcomes may be made between different biologics by class.

ISAR is open to other datasets that are not part of the core ISAR projects but have alignment of variables, which will enable combining data for specific projects. Thus, combining and collaborating with other databases or extracting data from other databases for specific projects is an available option.

## Discussion

ISAR is a global collaborative initiative that allows for prospectively and retrospectively analysing real-life severe asthma data at the patient-level. It is conducted by OPC, with academic and regulatory oversight from the ISC, academic support from REG, ethical governance from ADEPT and joint funding support from OPC and AstraZeneca. ISAR is facilitated by standard data collection via a core set of variables across all participating registries and supporting data collection via electronic data capture, hosting and data entry. Benefitting from the use of aggregate vs. individualised data, ISAR is large enough to provide sufficient statistical power to detect differences and trends. It thus enables collaborating registries to answer key research questions on asthma at a global scale.

**Fig. 6:**
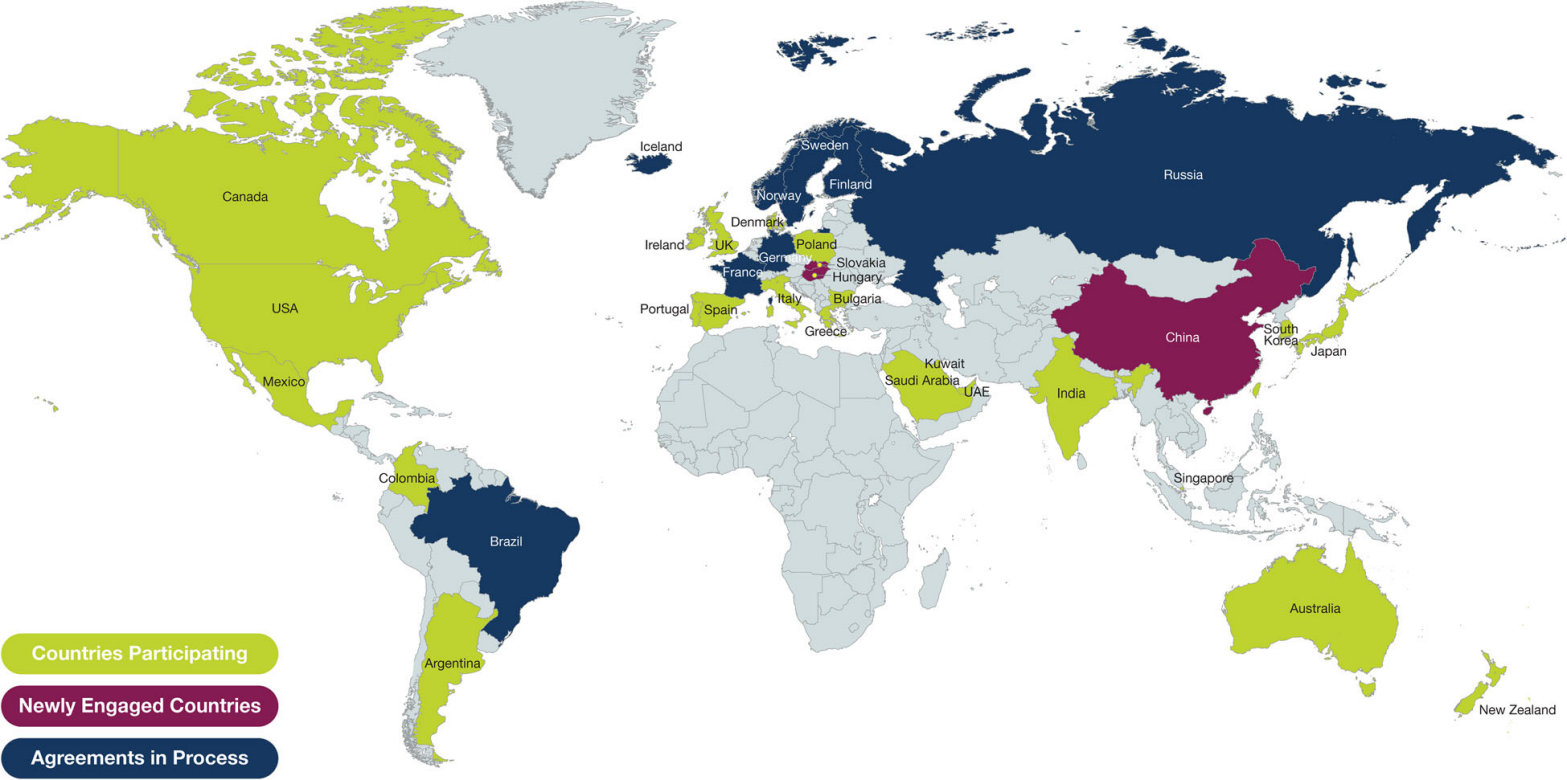
Current International Severe Asthma Registry snapshot^a^. ^a^Source: image created in-house. *UAE* United Arab Emirates, *UK* United Kingdom, *USA* United States of America

**Fig. 7:**
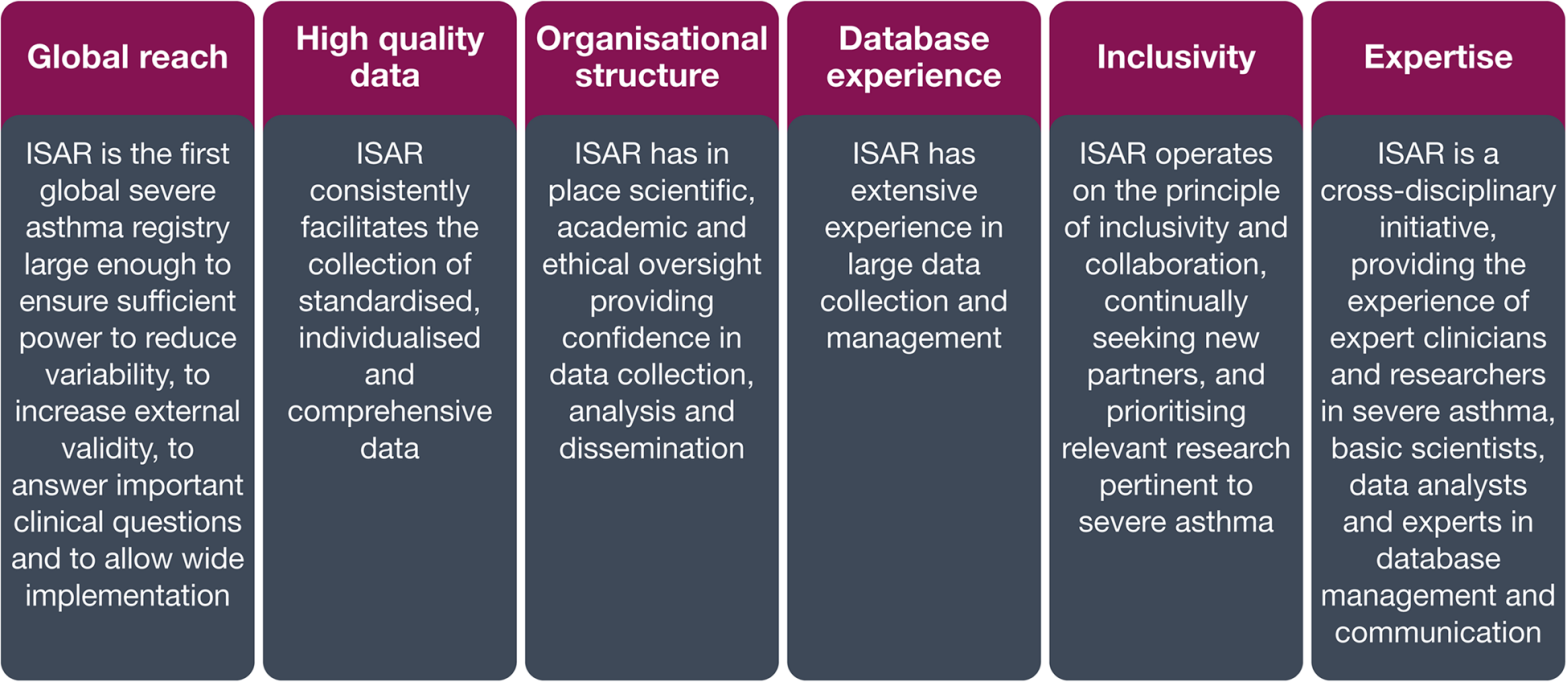
Key strengths of the International Severe Asthma Registry. *ISAR* International Severe Asthma Registry

Currently, ISAR’s membership includes registries from more than 30 participating countries (Fig. 6). The inclusive nature of ISAR will allow for extensive collaboration and potentially new research ideas. To ensure transparency, ISAR has in place a democratic voting system for selection of core research projects. In addition, data quality checks run by ISAR may lead to a robust dataset enabling quality research if countries decide to conduct their own research with their local data. Overall, ISAR aims to consolidate current knowledge of severe asthma based on six key strengths (Fig. 7), including its global reach, high quality data drawn from a large sample size, organisational structure, experience with database management inclusivity and expertise, enabling the generation of research that can have an impact on patient care globally.

Certain limitations of ISAR should be acknowledged. First, any database is only as good as the data it contains (i.e. what is measured, in whom, how and the extent of missing data). Second, due to their design, registry data may possess lower internal validity than data collected prospectively in randomised clinical trials, limiting the extent to which they can support causal relationships. The large volume of data also creates the potential for many analyses with selective reporting, although this will be mitigated by open disclosure of the research conducted on the dataset and the respective results. While ISAR has a broad representation from a number of countries, for some, there may be limited representation within the country itself. For example, ISAR currently covers only one site in the United States. Lastly, to become a truly global representation of severe asthma, funding issues need to be overcome to include patients from sub-Saharan Africa and other low-to-middle income countries.

The ISAR initiative has already led to the generation of key data in the severe asthma field. The research topic prioritised by the ISC in the inaugural year 2017 was the ‘Demographic and clinical characteristics of severe asthma patients worldwide’. Characteristics described included demographic distribution, medication, comorbidities, asthma control, blood eosinophil counts, immunoglobulin E (IgE) levels, lung function and health care resource use. In total, 4990 patients from five registries contributed to this first research project of ISAR [34]. In 2018, the global research project was on the characterisation and comparison of eosinophilic and non-eosinophilic phenotypes (*Manuscript under journal review*). The primary objective was to describe and compare the demographic and clinical features of eosinophilic compared with non-eosinophilic asthma phenotypes in an international cohort of adult patients with severe asthma. The broader definitions of type 2 vs. non-type 2 asthma [35] will also be examined. The core project prioritised by the ISC for 2019 is the ‘Comparative effectiveness across severe asthma biologic classes (anti-interleukin-5 vs. anti-IgE targeted therapy) in patients eligible for both modalities’. Furthermore, additional ISAR research projects are prioritised and are open to researchers to join and lead (Table 3). Overall, the clinical questions that ISAR plans to answer are varied and range from a description of the patient population to the assessment of any differences between disease subtypes, drivers of treatment switching, biomarker profiles and the development of a protocol to identify hidden severe asthma.

**Table 3:** Prioritised research projects for 2018, 2019 and 2020

Project	Investigator
**2018**	
Biologics in severe asthma: utilisation patterns, causes for discontinuation and switching and adverse outcomes	Professor Andrew Menzies-Gow (UK)
Hidden severe asthma patients in primary care vs. ISAR	Professor David Price (Singapore)
Relationship between socioeconomic status and asthma outcomes	Professor Liam Heaney (UK)
The impact of exacerbation burden on lung function trajectory in a broad asthma population and severe asthma population	Professor Liam Heaney (UK)
Biomarker Relatability in the International Severe Asthma Registry (BRISAR)	Dr. Eve Denton and Dr. Mark Hew (Australia)
Identification of predictors (i.e. biomarkers) of response to biologics	Dr. Eve Denton and Dr. Mark Hew (Australia)
Hidden chronic asthma within the COPD/ACO population	Professor Chin Kook Rhee (South Korea)
Age of onset of asthma in severe asthma patients	Dr. Enrico Heffler (Italy)
**2019**	
Describe the OCS landscape: annual consumption, prevalence, outcomes and side effects of long-term OCS users	
Criteria for choosing and switching between similar biological treatment options in patients with atopic and non-atopic severe eosinophilic asthma	
**2020**	
What is the impact of co-morbidity in severe asthma?	
Define responders and non-responders to biologics and describe their characteristics overall and per biologic	
Describe the clinical outcome before and after biologic treatment by biologic class, by individual biologic, and by subgroups of baseline characteristics	

*ACO* asthma-chronic obstructive pulmonary disease overlap, *COPD* chronic obstructive pulmonary disease, *FEV*_*1*_ forced expiratory volume in 1 second, *ISAR* International Severe Asthma Registry, *OCS* oral corticosteroids, *UK* United Kingdom

There is precedence for the use of global registries to facilitate international collaboration and contribute to our understanding of other diseases [36, 37]. For example, the Translational Research in Europe – Assessment & Treatment of Neuromuscular Diseases (TREAT-NMD), which is a network for the neuromuscular field, enabled harmonised implementation of registries featuring patients with Duchenne’s muscular dystrophy [36]. The European Register for Multiple Sclerosis (EUReMS) was created to enable comparisons across EU countries by merging data from existing national multiple sclerosis registries and regional cohorts [37]. In rheumatology, collaboration across global registries made access to rich data sources possible, enabling investigation of safety outcomes among a geographically diverse rheumatoid arthritis patient population [38]. Thus, ISAR has the potential to become an important platform to facilitate severe asthma research. Furthermore, the protocol described here can potentially be used as a foundation in other diseases, where divergent national and regional registries preclude collaboration and inter-operability between registries.

In the future, ISAR plans to include additional countries, covering Africa, Asia, South America, the Middle East and Eastern Europe. Other prospects include linkages with other databases and integration with electronic medical records. In addition, longitudinal research in patients with less severe asthma and the development of a paediatric ISAR in order to cover the entire severe asthma life cycle are also being considered.

## Conclusion

ISAR is the first global registry for adult severe asthma that captures a large volume of standardised, international data on severe asthma. By acting as a data custodian of international patient data, ISAR works as an open border initiative, providing a platform to facilitate data sharing. The registry provides enough statistical power to address important research questions in severe asthma aimed at a wide range of topics, including knowledge of patient presentations, disease heterogeneity and the natural history of severe asthma; diagnosis and disease stratification; identification of predictors of treatment success and new treatment targets; demonstration of how treatments are used in real-life and how effective they are; long-term safety in different patient populations and comparison of differences between countries and care systems. Through ISAR, it is expected that the harmonised, standardised nature of data contained and the collaborative partnerships being made possible may reveal previously unthought of or hitherto neglected research avenues.

## Supplementary information

**Additional file 1: Appendix.** International Severe Asthma Registry study group.

**Additional file 2: Table S1.** Full list of ISAR 95 core variables.

**Additional file 3: Table S2.** International Severe Asthma Registry bolt-on variables.

**Additional file 4: Table S3.** International Severe Asthma Registry optional additional research variables.

## Data Availability

Not applicable.

## Abbreviations

ACO: asthma-chronic obstructive pulmonary disease overlap
ACQ: Asthma Control Questionnaire
ACT: Asthma Control Test
ADEPT: Anonymised Data Ethics & Protocol Transparency
ATS: American Thoracic Society
eCRF: Electronic Case Report Form
EDC: Electronic Data Capture
ENCePP: European Network of Centres for Pharmacoepidemiology and Pharmacovigilance
ERS: European Respiratory Society
EU GDPR: European Union General Data Protection Regulation
EUReMS: European Register for Multiple Sclerosis
FEV_1_: forced expiratory volume in 1 s
FVC: forced vital capacity
GINA: Global Initiative for Asthma
ICS: inhaled corticosteroids
ICU: intensive care unit
IgE: immunoglobulin E
ISAR: International Severe Asthma Registry
ISC: ISAR Steering Committee
LABA: long-acting β_2_ agonist
OPC: Optimum Patient Care
REG: Respiratory Effectiveness Group
SNOMED CT: Systematized Nomenclature of Medicine Clinical Terms
SOP: standard operating procedure
TREAT-NMD: Translational Research in Europe – Assessment & Treatment of Neuromuscular Diseases
UK: United Kingdom
