# Supplementary material for: International severe asthma registry (ISAR): protocol for a global registry
Source: BMC Med Res Methodol. 2020 Aug 14;20:212. doi: 10.1186/s12874-020-01065-0 (PMC7439682; doi:10.1186/s12874-020-01065-0)
Supplement: Supplementary file 1 — Additional file 1: Appendix. International Severe Asthma Registry study group. [file 12874_2020_1065_MOESM1_ESM.docx]

**Additional file 1: Appendix**

**International Severe Asthma Registry study group**

- Alacqua, Marianna, MD, PhD; Global Medical Affairs, AstraZeneca, Gaithersburg, USA.
- Altraja, Alan, MD; Department of Pulmonary Medicine, University of Tartu & Lung Clinic, Tartu University Hospital, Tartu, Estonia.
- Backer, Vibeke, MD, DMSci; Center of Physical Activity Research, Rigshospitalet and Copenhagen University, Copenhagen, Denmark, Copenhagen, Denmark.
- Bel, Elisabeth, MD, PhD; Department of Respiratory Medicine, Academic Medical Centre, University of Amsterdam, The Netherlands.
- Bjermer, Leif, MD, PhD; Department of Respiratory Medicine & Allergology, Skåne University Hospital, Lund, Sweden.
- Bjornsdottir, Unnur, MD; Faculty of Medicine, University of Iceland, Reykjavik, Iceland.
- Bourdin, Arnaud, MD, PhD; Department of Respiratory Diseases, Montpellier University Hospitals, Hopital Arnaud de Villeneuve and PhyMed Exp (INSERM U 1046, CNRS UMR9214), Universite de Montpellier, Montpellier, France.
- Brusselle, Guy, MD, PhD; Department of Respiratory Medicine, Ghent University Hospital, Ghent, Belgium, and Departments of Epidemiology and Respiratory Medicine, Erasmus Medical Center Rotterdam, Rotterdam, The Netherlands.
- Buhl, Roland, MD, PhD; Pulmonary Department, Johannes Gutenberg University Mainz, Mainz, Germany.
- Bulathsinhala, Lakmini, MPH; Optimum Patient Care, Cambridge, UK.
- Busby, John, PhD; Centre for Public Health, Queen’s University Belfast, Belfast, UK.
- Canonica, Giorgio Walter, MD, PhD; Personalized Medicine Asthma & Allergy Clinic, Humanitas University & Research Hospital, Milan, Italy, and SANI-Severe Asthma Network Italy, Italy.
- Carter, Victoria, BSc; Optimum Patient Care, Cambridge, UK.
- Chaudhry, Isha, MSc; Optimum Patient Care, Cambridge, UK.
- Christoff, George, MD, PhD, MPH; Medical University – Sofia, Faculty of Public Health, Sofia, Bulgaria.
- Cho, You Sook, MD, PhD; Division of Allergy, Department of Medicine, Asan Medical Center, College of Medicine, University of Ulsan, Seoul, South Korea.
- Cosio, Borja G, MD, PhD; Respiratory Medicine, Son Espases University Hospital‑IdISBa‑Ciberes, Mallorca, Spain.
- Costello, Richard W, MD; Clinical Research Centre, Smurfit Building Beaumont Hospital and Department of Respiratory Medicine, RCSI, Dublin, Ireland.
- Eleangovan, Neva, BSc; Optimum Patient Care, Cambridge, UK.
- FitzGerald, J Mark, MD, FRCPC; The Institute for Heart Lung Health, Vancouver, Canada.
- Gibson, Peter G, MD; Australasian Severe Asthma Network, Priority Research Centre for Healthy Lungs, University of Newcastle, Newcastle, Australia, and Hunter Medical Research Institute, Department of Respiratory and Sleep Medicine, John Hunter Hospital, New Lambton Heights, Australia.
- Hardjojo, Antony, PhD; Optimum Patient Care, Cambridge, UK.
- Heaney, Liam G, MD; Queen’s University Belfast, Belfast, UK.
- Heffler, Enrico, MD, PhD; Personalized Medicine Asthma & Allergy Clinic, Humanitas University & Research Hospital, Milan, Italy, and SANI-Severe Asthma Network Italy, Italy.
- Hew, Mark, MD, PhD; Alfred Health & Monash University, Melbourne, Australia.
- Hirsch, Ian; PhD; Statistics and Biometrics, AstraZeneca, Gaithersburg, USA.
- Hosseini, Naeimeh, MD; Optimum Patient Care, Cambridge, UK.
- Iwanaga, Takashi, MD, PhD; Department of Respiratory Medicine and Allergology, Kindai University Hospital, Faculty of Medicine, Ōsakasayama, Japan.
- Jackson, David J, MD, PhD; Guy’s & St Thomas’ NHS Trust and King’s College London, London, UK.
- Jones, Rupert, MD; Faculty of Medicine & Dentistry, University of Plymouth, Plymouth, UK.
- Koh, Mariko Siyue, MRCP; Department of Respiratory & Critical Care Medicine, Singapore General Hospital and Duke-National University Singapore Medical School, Singapore.
- Le, Thao, BCom; Optimum Patient Care, Cambridge, UK.
- Lehmann, Sverre, MD, PhD; Department of Clinical Science, University of Bergen, and Department of Thoracic Medicine, Haukeland University Hospital, Bergen, Norway.
- Lehtimäki, Lauri, MD, PhD; Allergy Centre, Tampere University Hospital and Tampere University, Tampere, Finland.
- Ludviksdottir, Dora, MD, PhD; Department of Respiratory, Faculty of Medicine, Landspitali University Hospital and University of Iceland, Reykjavik, Iceland.
- Maitland-van der Zee, Anke-Hilse, MD; Department of Respiratory Medicine, Amsterdam AMC, University of Amsterdam, Amsterdam, The Netherlands.
- Menzies-Gow, Andrew, MD, PhD; UK Severe Asthma Network, Royal Brompton & Harefield NHS Foundation Trust, London, UK.
- Murray, Ruth B, PhD; Optimum Patient Care, Cambridge, UK.
- Papadopoulos, Nikolaos G., MD, PhD; University of Athens, Athens, Greece, and University of Manchester, Manchester, UK.
- Perez-de-Llano, Luis, MD, PhD; Pneumology Service, Hospital Universitario Lucus Augusti, Lugo, Spain.
- Peters, Matthew, MD, PhD; University of Sydney Medical School, Sydney, Australia.
- Pfeffer, Paul E, MRCP, PhD; UK Severe Asthma Network; Barts Health NHS Trust and Queen Mary University of London, London, UK.
- Plaza, Vicente, MD, PhD; Department of Respiratory Medicine, Hospital de la Santa Creu y Sant Pau, Barcelona, Spain; Institut d'Investigació Biomédica Sant Pau, IIB Sant Pau, Barcelona, Spain; and Department of Medicine, Universitat Autònoma de Barcelona, Barcelona, Spain.
- Porsbjerg, Celeste Michala, MD, PhD; Respiratory Research Unit, Department of Respiratory Medicine, Bispebjerg Hospital, Copenhagen University, Copenhagen, Denmark.
- Price, Chris, LLB; Optimum Patient Care, Cambridge, UK.
- Price, David B, MD, FRCGP; Optimum Patient Care, Cambridge, UK; Observational and Pragmatic Research Institute, Singapore; and Academic Primary Care, University of Aberdeen, Aberdeen, UK.
- Rhee, Chin Kook, MD, PhD; Division of Allergy, Pulmonary and Critical Care Medicine, Seoul St. Mary's Hospital, College of Medicine, The Catholic University of Korea, Seoul, South Korea.
- Sadatsafavi, Mohsen, MD, PhD; Faculty of Pharmaceutical Sciences, University of British Columbia, Vancouver, Canada.
- Taillé, Camille, MD, PhD; Assistance Publique-Hôpitaux de Paris, Hôpital Bichat, DHU FIRE, Service de Pneumologie, INSERM UMR1152, Université Paris Diderot, Labex Inflamex, Paris, France.
- Tohda, Yuji, MD, PhD; Department of Respiratory Medicine and Allergology, Kindai University Hospital, Faculty of Medicine, Ōsakasayama, Japan.
- Tran, Trung N, MD, PhD; Epidemiology, AstraZeneca, Gaithersburg, USA.
- Wang, Eileen, MD, MPH; Division of Allergy & Clinical Immunology, Department of Medicine, National Jewish Health, and Division of Allergy & Clinical Immunology, Department of Internal Medicine, University of Colorado Hospital, Denver and Aurora, Colorado, USA.
- Wechsler, Michael E, MD, MMSc; Division of Pulmonary, Critical Care and Sleep Medicine, Asthma Program, National Jewish Health, Denver, USA.
- Zangrilli, James, MD; AstraZeneca, Gaithersburg, USA.
