# Supplementary material for: International severe asthma registry (ISAR): protocol for a global registry
Source: BMC Med Res Methodol. 2020 Aug 14;20:212. doi: 10.1186/s12874-020-01065-0 (PMC7439682; doi:10.1186/s12874-020-01065-0)
Supplement: Supplementary file 2 — Additional file 2: Table S1. Full list of ISAR 95 core variables. [file 12874_2020_1065_MOESM2_ESM.docx]

**Additional file 2: Supplementary Table S1.** Full list of ISAR 95 core variables [1]

| **Category** | **Variable field name** | **Recorded units** |
| --- | --- | --- |
| **Patient details** | Date of visit | DD/MM/YYYY |
|  | Date of birth | DD/MM/YYYY |
|  | Age at assessment | Number (auto-calculated) |
|  | Gender | Female/male |
|  | Ethnicity | Caucasian/South East Asian/North East Asian/African/Mixed/Other |
|  | BMI, height, weight | Decimal numbers (auto-calculated) |
|  | Has the patient had bronchial thermoplasty | No/Yes |
| **Occupation** | Current occupation | Free text |
| **Medical history** | Current smoking status | Never smoked/ex-smoker/current smoker |
|  | Pack years | Number (auto-calculated) |
|  | Years since last smoked | Number |
|  | Age when patient’s asthma symptoms began | Number (whole years or months if <1 year) |
|  | Number of exacerbations requiring rescue steroids in the past 12 months | Number |
|  | Total number of episodes of invasive ventilation | Number |
|  | Total number of asthma-related A&E attendances in the past 12 months | Number |
|  | Total number of asthma-related hospital admissions in the past 12 months | Number |
| **Comorbidity** | Indication of eczema | Never/past/current |
|  | Indication of allergic rhinitis | Never/past/current |
|  | Indication of chronic rhinosinusitis | Never/past/current |
|  | Indication of nasal polyps | Never/past/current |
|  | Indication of atopic disease  Yes, if indicated for eczema  Yes, if indicated for allergic rhinitis | No/Yes (auto-populated) |
| **Blood/Sputum** | Highest blood eosinophil count within the past year | Decimal number |
|  | Date of the highest blood eosinophil count within the past year | DD/MM/YYYY |
|  | Was this the highest blood eosinophil count during an exacerbation event? | No/Yes |
|  | The highest blood eosinophil count within the past year and not during exacerbation | Decimal number |
|  | Date of highest blood eosinophil count within the past year and not during an exacerbation event | DD/MM/YYYY |
|  | Current blood eosinophil count | Decimal number |
|  | Date of current blood eosinophil count | DD/MM/YYYY |
|  | The highest sputum eosinophil count within the past year (percentage) | Decimal number |
|  | Date of the highest sputum eosinophil count within the past year | DD/MM/YYYY |
|  | IgE count | Decimal number |
| **Diagnostics** | Chest CT scan | Normal/Abnormal/Not done |
|  | Date of chest CT scan | DD/MM/YYYY |
|  | Bone densitometry (DEXA) | No/Yes |
|  | Date of bone densitometry (DEXA) | DD/MM/YYYY |
| **Lung function** | Pre-bronchodilator FEV_1_ | Decimal number |
|  | Post-bronchodilator FEV_1_ | Decimal number |
|  | Pre-bronchodilator FVC | Decimal number |
|  | Post-bronchodilator FVC | Decimal number |
|  | Predicted FEV_1_ | Decimal number (auto-calculated) |
|  | Pre-bronchodilator FEV_1_ (% predicted) | Decimal number (auto-calculated) |
|  | Post-bronchodilator FEV_1_ (% predicted) | Decimal number (auto-calculated) |
|  | Predicted FVC | Decimal number (auto-calculated) |
|  | Pre-bronchodilator FVC (% predicted) | Decimal number (auto-calculated) |
|  | Post-bronchodilator FVC (% predicted) | Decimal number (auto-calculated) |
|  | FEV_1_/FVC ratio pre-bronchodilator (%) | Decimal number (auto-calculated) |
|  | FEV_1_/FVC ratio post-bronchodilator (%) | Decimal number (auto-calculated) |
|  | PC20 methacholine/histamine test | No/Yes |
|  | Date of PC20 test | DD/MM/YYYY |
|  | PC20 test result | Decimal number |
|  | FeNO test | No/Yes |
|  | Date of FeNO test | DD/MM/YYYY |
|  | FeNO test result | Decimal number |
| **Allergen testing** | Environmental allergen test | Serum allergen test (CAP, ELISA, RAST)/SPT/not done |
|  | Serum allergy test: Positive to perennial allergen | No/Yes |
|  | Serum allergy test: Specify positive allergen and result | Dust mite (e.g. *D. pteronyssinus*)/grass mix/cat hair/mould mix/dog hair/Aspergillus/other (please specify) |
|  | Date of serum allergy test | DD/MM/YYYY |
|  | SPT: Positive to allergen | No/Yes |
|  | SPT: Specify positive allergen and result | Grass mix/trees/weed mix/Aspergillus/mould mix/dust mite/food mix/animal mix/cat hair/dog hair/other (please specify) |
|  | Date of SPT | DD/MM/YYYY |
| **Asthma control**^a^ | GINA Asthma Control Questionnaire |  |
|  | In the past 4 weeks, did the patient have: |  |
|  | Daytime symptoms more than twice per week | No/Yes |
|  | Any activity limitation | No/Yes |
|  | Any nocturnal symptoms/awakening | No/Yes |
|  | Reliever medication use more than twice per week | No/Yes |
|  | Lung function (PEF or FEV_1_) <80% of predicted or personal best | No/Yes |
| **Asthma medication** | Maintenance oral corticosteroids | No/Yes |
|  | Start date of oral corticosteroids | DD/MM/YYYY |
|  | ICS + LABA combination therapy | No  Budesonide + Formoterol  Fluticasone furoate + Vilanterol  Fluticasone propionate + Salmeterol  Fluticasone propionate + Formoterol  Mometasone + Formoterol  Beclomethasone + Formoterol  Other |
|  | Start/end date of ICS + LABA combination therapy | DD/MM/YYYY |
|  | ICS (only) | No  Triamcinolone acetonide  Mometasone furoate  Fluticasone propionate  Fluticasone furoate  Ciclesonide  Flunisolide  Budesonide  Beclomethasone dipropionate  Other |
|  | Start/end date of ICS (only) therapy | DD/MM/YYYY |
|  | LABA | No  Formoterol  Salmeterol  Indacaterol  Arformoterol  Olodaterol  Other |
|  | Start/end date of LABA therapy | DD/MM/YYYY |
|  | LAMA | No  Aclidinium  Tiotropium  Umeclidinium  Glycopyrronium  Other |
|  | Start/end date of LAMA therapy | DD/MM/YYYY |
|  | Theophyllines | No  Theophylline  Aminophylline  Other |
|  | Start/end date of theophylline therapy | DD/MM/YYYY |
|  | LTRA | No  Zafirlukast  Montelukast  Other |
|  | Start/end date of LTRA therapy | DD/MM/YYYY |
|  | Anti-IgE treatment | No/Yes |
|  | Start/end date of anti-IgE therapy | DD/MM/YYYY |
|  | Anti-IL-5/IL-5R treatment, other | No  Reslizumab  Mepolizumab  Benralizumab  Other^b^ |
|  | Start/end date of anti-IL-5 therapy | DD/MM/YYYY |
|  | Macrolide antibiotic treatment | No  Azithromycin  Clarithromycin  Erythromycin  Roxithromycin  Fidaxomicin  Telithromycin  Other |
|  | Start/end date of macrolide antibiotic therapy | DD/MM/YYYY |
|  | Other steroid-sparing agents | Free text |
| **Adherence** | Evidence of poor adherence^c^ | No  Yes: Clinical impression  Yes: Objective measures  Yes: Prescription records |
|  | Other factors contributing to severe asthma symptoms^d^ | Free text |
| **Management plan** | Current clinical management plane | Discharge to local service  Optimisation of current treatment  Biologic therapy (specific drug can be found in current medication)  Bronchial thermoplasty  Maintenance oral corticosteroids  Steroid-sparing agent (specific drug can be found in current medication)  Enter into clinical trial  Other (please specify) |

*A&E* accident and emergency, *BMI* body mass index, *CAP* ImmunoCAP test, *CT* computed tomography, *DEXA* dual energy X-ray absorptiometry, *ELISA* enzyme-linked immunosorbent assay, *FeNO* fractional exhaled nitric oxide, *FEV_1_* forced expiratory volume in 1 second, *FVC* forced vital capacity, *GINA* Global Initiative for Asthma, *ICS* inhaled corticosteroids, *IgE* immunoglobulin E, *IL-5* interleukin-5, *ISAR* International Severe Asthma Registry, *LABA* long-acting β_2_-agonist, *LAMA* long-acting muscarinic antagonist, *LTRA* leukotriene receptor antagonist, *PC20* provocative concentration of methacholine/histamine needed to produce a 20% decrease in FEV_1_, *PEF* peak expiratory flow, *RAST* radio-allergosorbent test, *SPT* skin prick test.

^a^Asthma Control Questionnaire or the Asthma Control Test are optional extras for this category (depending on registry preference).

^b^Other new biologics will be added once approved and in use.

^c^Poor adherence to treatment can be indicated by selecting either (a) or (b):

1. Clinical impression: Opinion of a medical personnel

E.g. i) Impression of ‘non-persistence’: Patient stops taking medication

ii) Impression of ‘non-conformation’: Patient does not take medication as prescribed

1. Prescription records: Evidenced by medical records detailing prescriptions being issued and inadequately filled

E.g. Medication possession ratio (MPR) = (Sum of days’ supply for all fills/Number of days) x 100% <80% threshold

^d^Calls for a trained clinician’s perception or opinion on any other external factors (if any) potentially contributing to the severe asthma symptoms.

E.g. Weather (cold air), air pollution, physical activity (exercise-induced asthma symptoms), occupational triggers (workplace irritants, gases, chemical fumes, dust), strong smells (perfumes), prior respiratory infections.

^e^Aims to record the asthma action plan for a patient to review efficacy over time.

E.g. i) Entry into clinical trial: If patient can benefit from a clinical trial drug

ii) Discharge to local asthma service: If patient has shown alleviated asthma symptoms

iii) Optimisation of current asthma therapy: If patient’s current asthma therapy is titrated for better asthma management

iv) Bronchial thermoplasty: If patient is eligible to have a surgery to manage their asthma

v) Biologic therapy: If patient is prescribed biologic therapy

vi) Others: Asthma education and inhaler use education

**Reference**:

1. Bulathsinhala L, Eleangovan N, Heaney LG, Menzies-Gow A, Gibson PG, Peters M, et al. Development of the International Severe Asthma Registry (ISAR): a modified Delphi study. J Allergy Clin Immunol Pract. 2019;7:578-88. e2
