# Supplementary material for: International severe asthma registry (ISAR): protocol for a global registry
Source: BMC Med Res Methodol. 2020 Aug 14;20:212. doi: 10.1186/s12874-020-01065-0 (PMC7439682; doi:10.1186/s12874-020-01065-0)
Supplement: Supplementary file 3 — Additional file 3: Table S2. International Severe Asthma Registry bolt-on variables. [file 12874_2020_1065_MOESM3_ESM.docx]

**Additional file 3: Supplementary Table S2**. International Severe Asthma Registry bolt-on variables

| **Category** | **Variables s** |
| --- | --- |
| **Safety** | |
| Severe infection | Infection type |
|  | Start and end dates |
|  | Outcome of infection |
|  | Site of infection |
| Malignancy | Malignancy history, type, stage, status and diagnosis confirmation |
|  | Start and end dates |
|  | Outcome of malignancy |
|  | Site of malignancy |
| Anaphylactic reaction | Likely exposure of the reaction |
|  | Time to reaction |
|  | Date of the reaction |
|  | Outcome of the anaphylactic reaction |
| **Effectiveness** | |
| Comorbidities | Osteoporosis |
|  | Osteoporosis: Start date |
|  | Circulatory system disease |
|  | Circulatory system disease: Type |
|  | Circulatory system disease: Start date |
|  | Glaucoma or cataract disease |
|  | Ocular disease: Type |
|  | Ocular disease: Start date |
|  | Obstructive sleep apnoea |
|  | Obstructive sleep apnoea: Start date |
|  | Renal failure |
|  | Renal failure: Start date |
|  | Depression |
|  | Depression: Start date |
|  | Anxiety |
|  | Anxiety: Start date |
|  | Type II diabetes mellitus |
|  | Type II diabetes mellitus: Start date |
|  | Peptic ulcer |
|  | Peptic ulcer: Start date |
|  | Pneumonia |
|  | Pneumonia: Start date |
| Dosage | Label dose for oral corticosteroids |
|  | Frequency for oral corticosteroids |
|  | Label dose for inhaled corticosteroids |
|  | Frequency for inhaled corticosteroids |
| Exacerbation history | Dates of exacerbations indicated |
|  | Type of rescue steroid used with label dose, frequency, start and end dates |
| Medication switching | Reason for switch in patient’s asthma medication/treatment |
