# Supplementary material for: International severe asthma registry (ISAR): protocol for a global registry
Source: BMC Med Res Methodol. 2020 Aug 14;20:212. doi: 10.1186/s12874-020-01065-0 (PMC7439682; doi:10.1186/s12874-020-01065-0)
Supplement: Supplementary file 4 — Additional file 4: Table S3. International Severe Asthma Registry optional additional research variables. [file 12874_2020_1065_MOESM4_ESM.docx]

**Additional file 4: Supplementary Table S3**: International Severe Asthma Registry optional research variables

| **Category** | **Variables** |
| --- | --- |
| Additional comorbidities | e.g. GERD, vocal cord dysfunction, COPD, bronchiectasis |
| Additional diagnostics | e.g. bronchoscopy, oesophageal pH monitoring test |
| Additional spirometry variables (lung function tests) |  |
| Additional asthma control | - Asthma Control Questionnaire (ACQ7) - Asthma Control Test (ACT) |
| Other asthma medication | - List of other respiratory drug prescriptions |
| Additional medical history | - Childhood disease questionnaire - Vaccination history |
| Occupation history | - Occupation history - Impact on work and lifestyle |
| Paediatric severe asthma | - List of paediatric severe asthma questions |
| Quality of life or depression and anxiety questionnaires | - Asthma Health Questionnaire–Japan (AHQ) - Asthma Quality of Life Questionnaire (AQLQ) - EuroQol five dimensions Questionnaire (EQ-5D) - St. George’s Respiratory Questionnaire (SGRQ) - Hospital Anxiety and Depression Scale (HADS) - Severe Asthma Questionnaire (SAQ) |
| Severe asthma biomarkers |  |

*COPD* chronic obstructive pulmonary disease, *GERD* gastroesophageal reflux disease.
